# Transcription Factors of CAT1, EFG1, and BCR1 Are Effective in Persister Cells of *Candida albicans*-Associated HIV-Positive and Chemotherapy Patients

**DOI:** 10.3389/fmicb.2021.651221

**Published:** 2021-08-24

**Authors:** Elham Aboualigalehdari, Maryam Tahmasebi Birgani, Mahnaz Fatahinia, Mehran Hosseinzadeh

**Affiliations:** ^1^Department of Medical Mycology, School of Medicine, Ahvaz Jundishapur University of Medical Sciences, Ahvaz, Iran; ^2^Department of Medical Genetics, School of Medicine, Ahvaz Jundishapur University of Medical Sciences, Ahvaz, Iran; ^3^Cellular and Molecular Research Center, Medical Basic Sciences Research Institute, Ahvaz Jundishapur University of Medical Sciences, Ahvaz, Iran; ^4^Infectious and Tropical Diseases Research Center, Health Research Institute and Department of Medical Mycology, School of Medicine, Ahvaz Jundishapur University of Medical Sciences, Ahvaz, Iran; ^5^Thalassemia and Hemoglobinopathy Research Center, Health Research Institute, Ahvaz Jundishapur University of Medical Sciences, Ahvaz, Iran

**Keywords:** oral candidiasis, persister cells, biofilm, HIV patients, patients under chemotherapy, *BCR1*, EFG1, *CAT1*

## Abstract

**Background:**

Biofilm is an accumulation of cells, which are formed on mucosal surfaces of the host as well as on medical devices. The inherent resistance of *Candida* strains producing biofilms to antimicrobial agents is an important and key feature for biofilm growth, which can lead to treatment failure. This resistance is due to the regulatory increase of the output pumps, the presence of extracellular matrix, and the existence of persister cells. Persister cells are phenotypic variants that have MICs similar to antibiotic-sensitive populations and are able to tolerate high doses of antibiotics. The current study investigated the possible role of *EFG1, BCR1*, and *CAT1* in the establishment or maintenance of persister cells in *Candida albicans* strains that produce biofilms.

**Methods:**

After identifying *Candida* isolates by molecular methods, *C. albicans* isolates were confirmed by sequencing. Isolation of persister cells and determination of their MIC were performed by microdilution method. Then, RNA extraction and cDNA synthesis were performed from 60 *C. albicans* isolates under promoting and inducing conditions. Afterward, the mean expression of *BCR1, EFG1*, and *CAT1* genes in both persister and non-persister groups was calculated using real-time qPCR. Phylogeny tree of persister and non-persister group isolates was drawn using ITS fragment.

**Results:**

A total of 77 persister isolates were taken from the oral cavity of HIV patients as well as from patients undergoing chemotherapy. Biofilm intensity in persister isolates separated from HIV-infected patients was different from the non-persister group. The mean fold change of *BCR1* (10.73), *CAT1* (15.34), and *EFG1* (2.41) genes in persister isolates was significantly higher than these genes in isolates without persister.

**Conclusion:**

It can be concluded that the most important factor in the production of persister cells is biofilm binding and production, not biofilm development or mature biofilm production, which was found in the expression of *BCR1* gene without change in the expression of *EFG1* gene in the persister group. Also, catalase plays an essential role in the production of persister in *C. albicans* biofilm producers with ROS detoxification.

## Introduction

Oral candidiasis is known as the most common fungal infection. It is an opportunistic disease among humans, especially in patients undergoing chemotherapy, transplant recipients, and HIV patients. Aging, uncontrolled diabetes mellitus, broad-spectrum antibiotics, corticosteroid, and/or immunosuppressant drug use are predisposing factors for this disease. It acts also as a prognostic marker for systemic diseases such as diabetes mellitus and a common problem in immunocompromised patients such as HIV patients and those undergoing chemotherapy (Aboualigalehdari et al., 2013, 2020; Taff et al., 2013; Cavalheiro and Teixeira, 2018; Putranti et al., 2018).

Studies have indicated that this disease occurs in about 80–90% of HIV-positive patients and also in 7–52% of patients undergoing chemotherapy. This infection is often detected in these patients in chronic and recurrent forms, especially in HIV-positive patients and leads to esophageal candidiasis and subsequent difficulty in digesting and swallowing (Jayachandran et al., 2016; Patil et al., 2018).

*Candida albicans* is the most important causative agent of oral candidiasis as well as an opportunistic organism that exists as normal microflora on the skin and mucous membranes of the body. This organism causes superficial to systemic infections, and the mortality rate due to infections caused by this organism is reported to be 40%. The prevalence of *C. albicans* isolated orally from patients with leukemia undergoing chemotherapy is about 46.2%, and in AIDS patients, it has been reported to be 37.2–95.2% (Piekarska et al., 2008; Li et al., 2015; Patil et al., 2018; Wuyts et al., 2018).

One of the important features in the pathogenicity of *C. albicans* is its ability to adhere and subsequently form a biofilm on biotic and abiotic surfaces such as mucosal surfaces as well as implanted medical devices. Biofilm formation is among the factors of resistance to antifungal drugs, which leads to treatment failure and disease recurrence. Several phenomena are implicated in biofilm resistance, including increased metabolic activity, production of extracellular biofilm matrix, cell density, upregulation of drug efflux pumps, persister cells, and stress responses (Piekarska et al., 2008; Li et al., 2015; Wuyts et al., 2018; Galdiero et al., 2020).

Another problem is the failure in the treatment of *C. albicans* infection leading to chronic infections. Recently, persister cells have attracted attention as a reason for drug tolerance in *C. albicans*. Persister cells are a special phenotypic type of biofilm population making up a small part of the biofilm population, which are capable of tolerating high doses of anti-fungal therapy. Thus, when a population of persister cells is exposed to a high dose of anti-fungal drugs, a small number of these cells survive, and interestingly, if they are re-cultured and re-exposed to a high dose of anti-fungal drugs, the fungi show a similar reaction. To our knowledge, many studies have indicated failure of anti-fungal drugs in *C. albicans*, which could be due to the presence of persister cells (Clinical and Laboratory Standards Institute [CLSI], 2008; Silva et al., 2012; Maheronnaghsh et al., 2020). For this purpose, this study was performed on fungal pathogens that had MIC associated with amphotericin B-sensitive populations and were exposed to very high doses of amphotericin B. The aim of this study was to better understand fungal persister cells for comparing biofilm intensity and expression of genes involved in biofilm production pathway (*BCR1* and *EFG1*) as well as oxidative stress response pathway (*CAT1*) in persister cells of *C. albicans* isolates taken from patients with HIV who underwent chemotherapy.

## Materials and Methods

In this study, 201 HIV patients and 200 cancer patients undergoing chemotherapy satisfying baseline criteria were studied with ethical code# IR.AJUMS.REC.1397.894.

### Inclusion Criteria

1. Samples were isolated from patients who had one of the following symptoms: Patients who had inflamed lesions in the oral mucosa and on the tongue with red flakes, those who had a false white membrane in their oral cavity or creamy to white plaques, and patients whose sense of taste had changed or had dry mouth.
2. After culturing the oral swab on CHROMagar^TM^ *Candida* media, the number of colonies was counted and the grown samples were entered into the project with ≥ 10 colonies as patients colonized with *Candida* (Erköse and Erturan, 2007).
3. Confirmation of *C. albicans* samples was molecular and macroscopic.
4. Insertion of persister *C. albicans* isolates with MIC related to amphotericin B-sensitive population (MIC < 2 μg/ml) as well as non-persister *C. albicans* (Clinical and Laboratory Standards Institute [CLSI], 2008; Maheronnaghsh et al., 2020).

### Sample Collection and Phenotypic Identification

The samples taken from the patients were transferred to the Department of Medical Mycology, School of Medicine, Ahvaz Jundishapur University of Medical Sciences, Ahvaz, Iran. Then, the samples were cultured on CHROMagar^TM^ *Candida* media (CHROMagar^TM^, Pioneer, Paris, France) and incubated at 35°C for 48–72 h. In the next step, the grown clinical isolates were compared and identified in terms of colony color based on the desired culture medium brochure and standard samples. Then, the culture medium was examined for colony diversity and the colonies were counted based on colony-forming unit/swab. After that, the isolates were purified on Sabouraud Dextrose Agar + Chloramphenicol (SC) (Liofilchem, Italy). The medium and the isolates were transferred to microtubes containing sterile distilled water for long-term storage (6 months) at two temperatures: 30°C and room temperature.

### DNA Extraction

The genomic DNA was directly extracted by boiling method (Yamada et al., 2002; Silva et al., 2012).

### PCR-RFLP, Sequencing, and Duplex PCR

To confirm the isolates as *C. albicans*, PCR-RFLP, sequencing, and duplex PCR were performed. The primers for PCR-RFLP are listed in Table 1, and the enzyme for PCR-RFLP was *Msp*I. Sequencing was done after PCR of V9g and LS266 area as shown in Table 1.

**TABLE 1:** Primers for PCR-RFLP, sequencing, and duplex PCR.

**Genes**	**5′ to 3′**	**Method**	**References**
ITS1	Forward: TCC GTA GGT GAA CCT TGC GG	PCR-RFLP	Abraham et al., 2020
ITS4	Reverse: TCC TCC GCT TAT TGA TAT GC	PCR-RFLP	Abraham et al., 2020
CAL	Forward: TGGTAAGGCGGGATCGCTT	duplex PCR	El-Baz et al., 2021
	Reverse: GGTCAAAGTTTGAAGATATAC		
CDU	Forward: AACTTGTCACGAGATTATTTTT	duplex PCR	El-Baz et al., 2021
	Reverse: AAAGTTTGAAGAATAAAATGGC		
V9g	Forward: TTACGTCCCTGCCCTT TGTA	PCR	Li et al., 2018
LS266	Reverse: GCATT CCCAAACAACTCGACTC	PCR	Li et al., 2018

### Biofilm Formation Assay

Biofilm production was performed on RPMI 1640 medium. All isolates were cultured in Sabouraud Dextrose Broth and incubated in a shaker incubator at 30°C for 24 h. After 24 h, the grown colonies were washed twice with sterile phosphate buffer solution (1× PBS) (pH 7.4) (Sigma-Aldrich) and centrifuged at 6063 rpm for 3 min at 4°C (SIGMA 1-15PK). Then, a yeast suspension was prepared from colonies with a concentration of 10^6^ cfu/ml in RPMI 1640 medium. Subsequently, in each well of flat bottom 96 microplates, 200 μl of suspension was poured in the wells. Culture medium was considered as the negative control and culture medium plus yeast suspension was the positive control. The microplates were placed at 37°C for 48 h. After this period, the culture medium in each well was emptied, and the wells were washed three times with sterile PBS. The microplates were then inverted at room temperature for an hour to dry. One hundred microliters of 0.1% crystal violet solution was added to each well and the microplate was placed at 37°C for 15 min without moving. Then, it was removed from the wells and washed again with sterile PBS, and the microplates were dried at room temperature. In the next step, 100 μl of 96% ethanol was poured into each well and the microplate was gently shaken in a circle by hand to extract the crystal violet color bound to the yeasts forming the biofilm faster. Then, the optical density (OD) of this solution was measured at 595 nm with a microplate reader (BioTek, Elx808). Finally, the biofilms were classified using the following formula (Abraham et al., 2020; El-Baz et al., 2021):

No biofilm: Absorbance ≤ Absorbance Control

Weak biofilm: Ac < A ≤ (2 × Ac)

Moderate biofilm: (2 × Ac) < A ≤ (4 × Ac)

Strong biofilm: (4 × Ac) < A

The isolates were grouped based on OD value. The isolates with low biofilm formation (LBF) were classified as first quarter (Q1); those with greater OD values were defined in the third quarter, which had high biofilm formation (HBF) (Q3), and the isolates with OD values between the first and third quarters were classified as second quarter and possessed intermediate biofilm formation (IBF) (Q2) (Li et al., 2018).

### Persister Cell Assay

All the isolates were cultured on Sabouraud Dextrose Broth and incubated in a shaker incubator at 30°C for 24 h. After this time, the grown colonies were washed twice with sterile PBS and centrifuged at 6063 rpm for 3 min at 4°C. A yeast suspension with a concentration of 10^6^ cfu/ml was subsequently prepared from colonies in RPMI 1640 medium. One hundred microliters of the prepared yeast suspension was added to each well of 96-well flat bottom microplate, which was placed at 37°C for 4 h. After this period, the culture medium in each well was discarded and washed with PBS, 100 μl of RPMI1640 culture medium was added to the wells and incubated for 24 h at 37°C. The culture medium in each well was emptied, and 200 μl of amphotericin B (Sigma Aldrich-USA) diluted with RPMI1640 with a concentration of 100 μg/ml (Moazeni et al., 2014; De Brucker et al., 2016; Alonso et al., 2018) was added to the wells and placed at 37°C for 48 h, after which the contents of each well were discarded and washed once with 100 μl of PBS. After washing, 100 μl of PBS was added to each well and homogenized using a sterile pipette. Twenty microliters from each well was serially diluted in 1:10 ratio in 10 dilutions of PBS. After dilution, 50 μl of each well was harvested and cultured on yeast peptone dextrose agar or YPD medium (Liofilchem, Italy). The plates were kept in an incubator at 48°C for 48–72 h. The plates were examined for colony count after 48 h and the samples were reported in three categories: high persister, low persister, and non-persister (LaFleur et al., 2006, 2010). Finally, samples containing persister cells were examined for MIC determination of amphotericin B according to CLSI M27-S4 guidelines, and if they were sensitive to the population with MIC < 2 μg/ml, the samples were confirmed as persister isolates (Clinical and Laboratory Standards Institute [CLSI], 2008; Maheronnaghsh et al., 2020).

### Evaluation of the Genes Responsible for Biofilm Formation and Oxidative Stress Among Isolates Producing Persister Cells

To determine the maximum and minimum survival rates, the isolates were ranked from highest to lowest based on their survival rates against amphotericin and then the first 15 isolates with the highest survival rates were specified as the persister group and the last 15 isolates not showing survival were chosen as those without persister or non-persister group. There were a total of 30 isolates in these two groups. Evaluation of *BCR1, EFG1*, and *CAT1* gene expression was performed in 30 *C. albicans* isolates by the real-time qPCR method. Real-time qPCR was done on isolates that were under inducing and promoting conditions.

Inducing conditions were applied for isolates in the state of biofilm production while promoting conditions were meant for isolates in the normal condition, and RNA was extracted from these isolates. In other words, the promoting conditions are the conditions before the formation of biofilm, and the inducing conditions are those after the formation of biofilm. Promoting conditions mean the alteration in expression of genes that cause biofilms and inducing conditions are changing the expression of genes causing biofilm formation to give specific properties to cells.

For this purpose, RNA was first extracted (RNX-Plus) and then the purity and integrity of extracted RNA were investigated under both conditions by nanodrop and loading of RNA on agarose gel, after which cDNA was synthesized from RNA (BioFact, South Korea). Finally, real-time qPCR was performed using Real-Time PCR Roche lightcycler^®^ 96, BioFACT^TM^ 2X Real-Time PCR Master Mix, and the primers listed in Table 2. Real-time qPCR was carried out with the following reaction conditions: 40 cycles of denaturation: 15 s at 95°C; annealing: 60 s at 60°C; and extension: 60 s at 72°C.

**TABLE 2:** qPCR primers.

**Genes**	**Primer sequence (5′→3′)**	**Size (bp)**	**Gene Bank**	**GC%**	**TM**	**References**
*BCR1*	Forward CTTCAGCAGCTTCATTAACACCTA	101	NC_032096.1	41.7	68	LaFleur et al., 2010
	Revers TCTTGGATCAGGTGTACTTTTCAA			37.5	66	
*EFG1*	Forward TGCCAATAATGTGTCGGTTG	100	XM_709144.2	45	58	Nikoomanesh et al., 2016
	Revers CCCATCTCTTCTACCACGTGTC			54	68	
*CAT1*	Forward GACTGCTTACATTCAAAC	117	NC_032089.1	38.9	55.1	Moazeni et al., 2014
	Revers AACTTACCAAATCTTCTCA			31.9	55.1	
*ACT1*	Forward ACTGCTTTGGCTCCATCTTCT	166	XM_019475182.1	48	65	
	Revers TGTGGTGAACAATGGATGGAC			48	62	

Primer efficiency was determined for all the gene expression assays using a standard curve and LinRegPCR software. The absence of dimer primers and contamination was controlled by observing the melting curves and loading products on the agarose gel. The absence of DNA was also checked with no RT control (no reverse transcriptase control). The expression level of *EFG1, BCR1*, and *CAT1* target genes in the isolates relative to that of the reference *ACT1* gene was first calculated manually by Excel and then confirmed by REST2009 software to ensure the accuracy of results. Finally, statistical analysis and plotting with SPSS 22 and GraphPad prism were done.

### Sequencing and Comparison of Persister and Non-persister Cells Using ITS Fragment

After sequencing of both groups, a software was used to draw the phylogenetic tree^1^, and the phylogenetic tree of *C. albicans* isolates was used using Maximum Likelihood and Boot Strap 100 to ensure the validity and reproducibility of the drawn trees. The table of genetic distances was drawn using MEGA 7 program of Pairwise Distances model. Then, cluster and clade were determined in the phylogenetic tree.

### Statistical Analysis

In this study, *t*-test and one-way ANOVA, Fisher’s exact test, and Mann–Whitney test with a significance level of <0.05 were used. The normality of data was checked using Shapiro–Wilk test. All tests were analyzed by SPSS software version 22.

## Results

A total of 104 *C. albicans* were isolated. Forty-three isolates were taken from patients undergoing chemotherapy and 61 *C. albicans* isolates were identified in HIV patients. All the isolates were confirmed by phenotypic and molecular methods to be *C. albicans*. Afterward, 104 *C. albicans* isolates, including isolates collected from HIV-infected patients and those undergoing chemotherapy, were examined to determine the severity of biofilm formation.

### *C. albicans* Is a Strong Biofilm Producer

**FIGURE 1:**
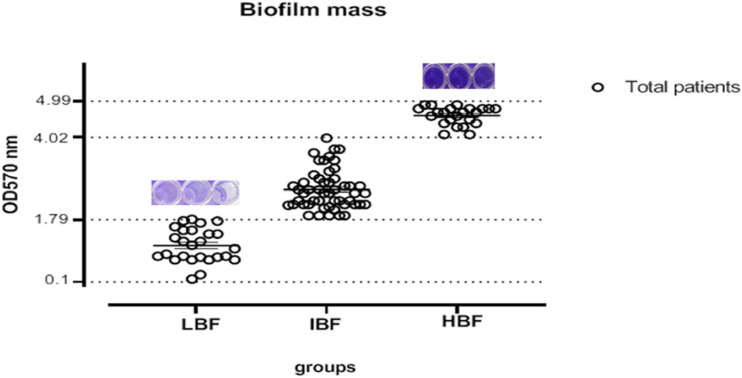
Determination of biofilm production level based on OD quarter; 25% of the isolates had 1.79 ≤ OD (LBF), 50% had 2.5 ≤ OD (IBF), and 75% of the isolates had 4.9 ≤ OD (HBF). LBF, low biofilm formation; IBF, intermediate biofilm formation; HBF, high biofilm formation.

Our analysis demonstrated that two isolates had a weak biofilm formation while 102 *C. albicans* isolates showed strong biofilm production. Another classification was also performed based on optical observation of the isolates that were grouped to three quarters as shown in Figure 1.

### The Prevalence of *C. albicans* Persister Cell Producers Is High Among *C. albicans* Population

**FIGURE 2:**
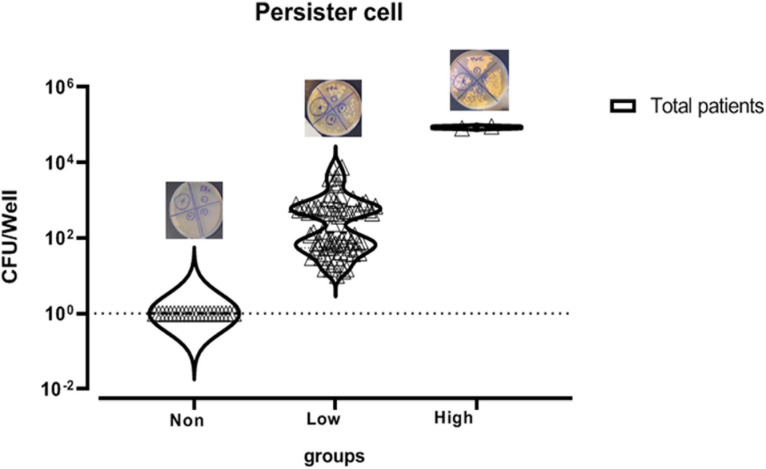
Mean survival rate or persister cell of *Candida albicans* isolates based on logarithmic data.

In this study, 104 *C. albicans* isolates were tested to separate persister cells from biofilm-forming isolates. This stage of persister cell isolation was done in triplicate and the results of each sample were reported as average. Out of 104 isolates under study that were exposed to 100 μg/ml amphotericin B, 83 had a survival rate in the range of 0.0003–9% and 21 had no survival ability in the face of the drug. Those isolates that were able to survive in the presence of amphotericin B were examined for MIC. To determine the sensitivity of the population, MICs of the isolates were read by microdilution method in the presence of amphotericin B in the range of 0.3–2 μg/ml. According to available guidelines, namely, CLSI M27-S4, out of 83 isolates, 77 with MIC < 2 μg/ml were sensitive and six isolates with MIC ≥ 2 μg/ml were classified as the resistant population and were excluded from the study. Therefore, 77 *C. albicans* were considered as persister cell isolates (Table 3 and Figure 2).

**TABLE 3:** The persister situation in infected patients with *C. albicans*.

**Persister cell (survival rate)**			
**Patients**	**High-persister**	**Low-persister**	**Non-persister**

Cancer patients (39)	1 (8%)	26 (0.0003–0.75%)	12
HIV patients (59)	1 (9%)	49 (0.0003–0.75%)	9
Total patients (98)	2 (8–9%)	75 (0.0003–0.75%)	21
Total patients (100%)	2.04%	76.5%	21.42%

According to a study by LaFleur et al. (2010), the isolates with a survival rate of >6% were defined as high persister, and those showing survival <6% were classified as low-persister cells. Besides, the isolates that had no survival were defined as non-persistent (Cohen et al., 2013).

Out of 77 *C. albicans* persister cells, two isolates with 9% and 8% survival were classified in the high-persister cell group, 75 isolates with the ability to survive in 0.75–0.0003% were classified in the low-persister group, and the 21 remaining isolates were classified in the non-persister group. Two high-persister isolates were taken from patients as follows: one was related to a patient undergoing chemotherapy who was hospitalized for approximately 2 months and did not have clear oral candidiasis symptoms. The only symptoms were dry mouth and redness of the tongue. Another isolate was taken from an HIV-infected patient recently discharged from the infectious ward of Razi Hospital who had been hospitalized for approximately 45 days. He had redness, inflammation, dry mouth, change in taste, and burning sensation, and of course, was in the end stage of the disease or so-called AIDS. According to the case file in Razi Hospital, only fluconazole was used for the patient whose disease was chronic and recurrent (Table 4).

**TABLE 4:** Information from two patients with high persister cells isolated from *Candida albicans*.

	**Age**	**Candida-load**	**Viral-load**	**CD4-count**	**Type of cancer**
				**cells/mm^3^**	
High persister (patient under chemotherapy)	75	20	–	–	Lung
		Colonization			
High persister (HIV patient)	45	10^5^	3,069,499	70	–
		Infection			

**FIGURE 3:**
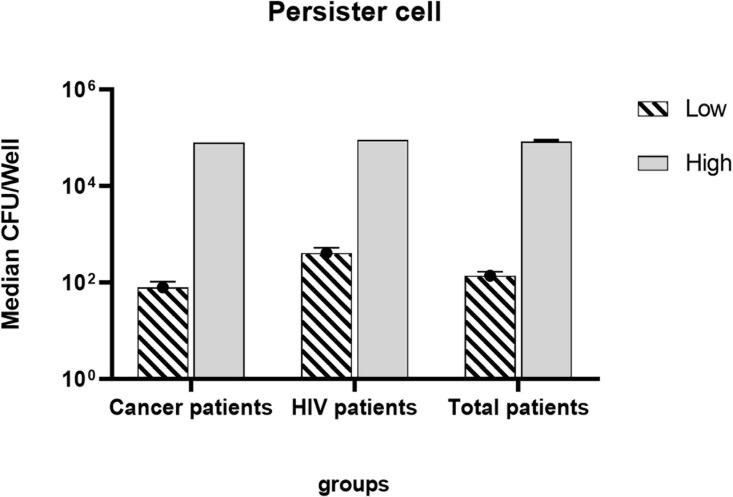
Log mean persister production in patients undergoing chemotherapy and HIV patients.

The mean production of low persister based on survival rate in HIV-infected patients with the ability of 0.04 ± 0.14% (survival rate ± standard deviation) was almost 5.5 times higher than patients undergoing chemotherapy with the ability of 0.008 ± 0.13%. The mean of low persister production in all patients was 0.01 ± 0.0013%. Since the survival rate and the number of high persisters in patients under study were almost the same, the same mean value was observed for both groups of patients (Figure 3).

### Comparison of Biofilm Intensity With Persister Cells in *C. albicans*

In this part of the research, we first deal with the relationship between different variables related to HIV patients with those related to patients undergoing chemotherapy with the production of persister cells, and then this variable is examined in all patients.

### HIV Patients

In this study, because there was only one isolate from HIV patients with a survival rate of 9%, statistical analysis was performed only in low and non-persister categories. Statistical results showed no significant correlation between qualitative variables of gender, tuberculosis, pneumocystosis, hepatitis B, hepatitis C, receiving antiviral drugs, tuberculosis prophylaxis drugs, HIV transmission from mother to child or through occupational exposure, blood transfusion, CD4 count, homosexuality, common syringe use for injection, and history of drug injection in non- and low-persister cells (Supplementary Table 1).

Data related to the age of subjects were normal, and no significant difference was observed between the two groups (low- and non-persister cells). There was no significant difference between viral and *Candida* loads between the two groups of non- and low-persister cells. However, the results of statistical tests showed that the biofilm variable is significantly different in the two groups of non- and low-persister cells (Supplementary Table 2) (*p* = 0.021).

### Patients Undergoing Chemotherapy

Considering that there was only one isolate in this group with a survival rate of 8% among the high-persister group, the statistical analysis was performed for two groups: low- and non-persister cells. Accordingly, the calculations showed that there was no significant relationship between gender and persister values in the non- and low persister cell groups.

Data related to the age of subjects were normal and no significant difference was observed between the two groups. Statistical results also showed no significant difference in *Candida* load and biofilm variables between the two groups (Supplementary Table 3).

**FIGURE 4:**
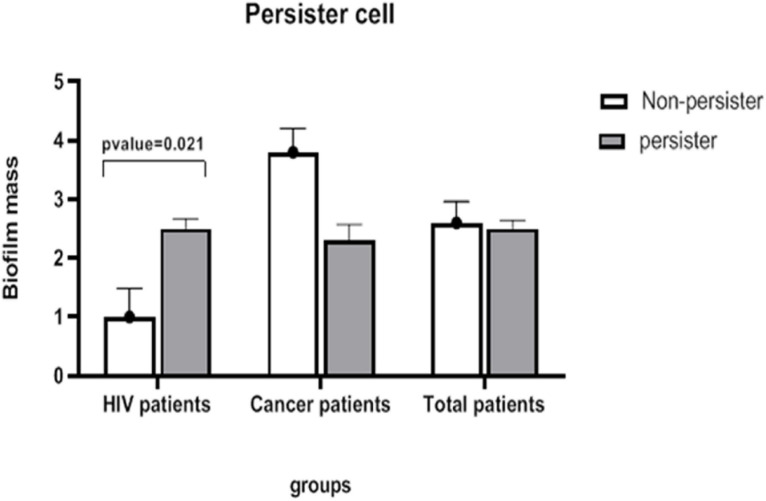
The mean severity of biofilm in the groups of persister and non-persister cells in patients with HIV and those undergoing chemotherapy. A significant relationship between persister cells and biofilm intensity is seen only in HIV-infected patients (*t*-test, *p*-value < 0.05).

Statistical analysis was performed using *t*-test in the two groups of non- and low-persister cells, but no significant difference was observed between the two groups (Figure 4).

### The Higher Expression of *EFG1, BCR1*, and *CAT1* in *C. albicans* Isolates Producing Persister Cells

Real-time qPCR was performed in promoting and inducing conditions. The purity of RNA on the A260/A280 and A260/A230 ratios was 1.8–2.0 and 1.9–2.0, respectively. Then, for the synthesis of cDNA, 500 ng of RNA entered the reaction.

### Promoting Condition

After normalizing the data with reference or control gene (*ACT1*), our findings showed an increase in the expression of *EFG1, BCR1*, and *CAT1* genes in persister isolates, among which the highest expression was related to *CAT1* gene, followed by *EFG1* and *BCR1*, respectively.

The mean expression of *EFG1, BCR1*, and *CAT1* genes was statistically evaluated using logarithmic data between the two groups of persister and non-persister isolates using *t*-test. Among the genes, only the expression of the *CAT1* gene was significant between persister and non-persister isolates (Supplementary Figure 1).

### Inducing Condition

In inducing conditions, the expression of *EFG1, BCR1*, and *CAT1* genes was evident in all isolates of both persister and non-persister groups. After normalizing the data with reference or control gene of *ACT1*, our results showed an increase in the expression of *EFG1, BCR1*, and *CAT1* genes in persister isolates under inducing conditions, among which the highest expression was related to *CAT1* gene, then *BCR1* and *EFG1*, respectively. The mean expression of *EFG1, BCR1*, and *CAT1* genes was statistically evaluated through logarithmic data between the two groups of persister and non-persister using *t*-test. The increasing expression of *BCR1* and *CAT1* was significant between the persister and non-persister groups (*p* < 0.05) (Supplementary Figure 1).

**FIGURE 5:**
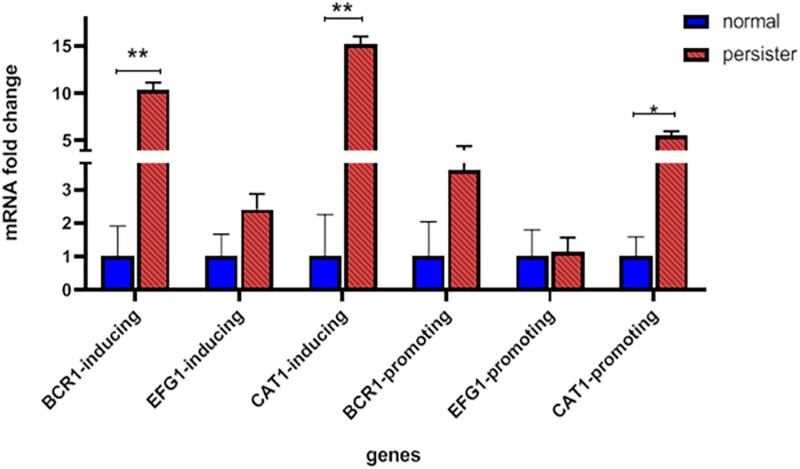
Fold change of 2^–ΔΔCT^ related to *CAT1, BCR1*, and *EFG1* genes in persister and non-persister isolates in inducing and promoting conditions (*t*-test, *p-value <0.05, **p-value <0.01).

The mean increase in gene expression under promoting and inducing conditions is described as fold change (i.e., 2^–ΔΔCT^). The highest amount of fold change in both promoting and inducing conditions was related to the *CAT1* gene with 15.34 and 5.54, respectively. The lowest fold change in promoting and inducing conditions was related to *EFG1* gene and its amount was 2.18 and 1.14, respectively (Table 5 and Figure 5).

**TABLE 5:** Comparison of mean fold change (2^–ΔΔCT^) in *EFG1*, *BCR*, and *CAT1* genes under two conditions: promoting and inducing.

	***BCR-P***	***BCR-I***	***EFG-P***	***EFG-I***	***CAT-P***	***CAT-I***
Persister	3.58 ± 0.25	10.73 ± 0.4	1.14 ± 0.15	2.41 ± 0.49	5.54 ± 0.53	15.34 ± 0.69
Normal	1	1	1	1	1	1
*P*-value (<0.05)	0.08	0.008*	0.1	0.1	0.03*	0.016*

SD, standard deviation; P, promoting; I, inducing (*t*-test,*P-value < 0.05).

### Phylogenetic Tree in Persister and Non-persister Groups by ITS Component

The DNA sequencing analysis of 20 isolates confirmed the presence of *C. albicans* and was recorded on DNA Data Bank of Japan (Accession No. LC612887-612906). After blasting, the similarity of sequences was reported to be 94–99.88%. The bootstrapping value was repeated 100 times to draw the phylogenetic tree to ensure the accuracy of drawing the phylogenetic tree. In our study, bootstrapping equal to 100 was reported, indicating that the phylogenetic tree of the desired nucleotides had a high degree of reliability. The similarity and intra-species difference of the sequences is calculated through pairwise distance. The average pairwise distance in sequences with a distance scale of 0.01 was 0.029. In these isolates, the biggest difference was related to 1A–low persister and 5A-normal isolates, which was equal to 8%. Then, the difference was related to 1A–low persister with normal 3A, 7A, and 8A isolates with a value of 7.8%. The isolate 6A–high persister, 5A-normal, and then 7A, 8A, and 3A had the highest difference (approximately 7%). The 6A–high-persister isolate had a genetic distance of 5.7% with the 7C–high-persister isolate.

**FIGURE 6:**
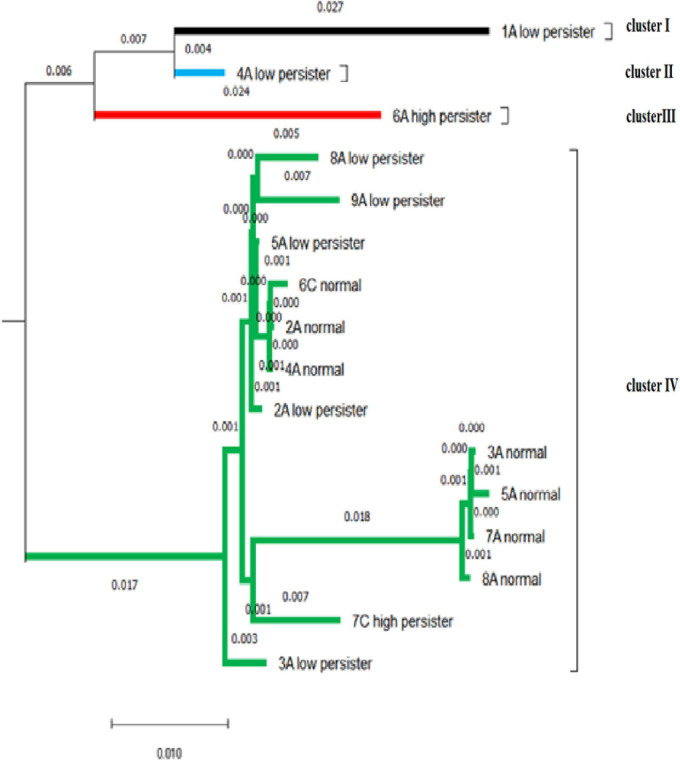
Based on the mean pairwise distance of 0.029, the phylogenetic tree of the isolates shows four clusters.

Based on the mean pairwise distance of 0.029, the sequences were divided into four clusters that were more genetically similar. The largest cluster belongs to cluster IV, which contains persister cell isolates and non-persister ones. Both high-persistent isolates were genetically separated by 5.7% in two separate clusters, and their offspring were completely different. 7c–high persister had no significant difference from the normal population, including 8A, 7A, 5A, 3A, 4A, 2A, and 6C by about 1–2% and was probably derived from the normal population. Most low-persister isolates differ by about 1% from the population of normal isolates, indicating that the nucleotide changes were negligible (Figure 6).

## Discussion

Over the past 30 years, advances in medical science have caused a significant increase in life-threatening candidiasis, which has a high prevalence and mortality associated with invasive candidiasis infections, despite progress in fungal drugs (Costa-de-Oliveira and Rodrigues, 2020). Today, despite drug susceptibility tests and appropriate treatments, comprehensive medicine has faced the phenomena of treatment failure and recurrence of the disease in patients. It is a concern in medical science to treat a patient with appropriate antifungal medication without failure in antifungal therapy. In fungi and bacteria, this likelihood increases such that populations of drug tolerance and persister cells may be overlooked, leading to treatment failure (Lewis, 2005).

The present study suggests that persister cells are present in *C. albicans* clinical isolates. Populations with high persister (above 6% survival) and low persister (0.0003–0.75%) were observed in the two groups of patients (i.e., HIV patients and those under chemotherapy). High-persister isolates were taken exclusively from patients who had been suffering from oral candidiasis for a long time and shared receiving antifungal drugs and long hospital stays. Also, based on statistical analysis, the total information of patients from whom a high persister cell was isolated proved that the persister cell isolate has no connection with *Candida* load of patients at the time of sampling; in other words, it is likely not related to the presence or absence of obvious oral candidiasis symptoms in the patient (Wuyts et al., 2018). Similar to LaFleur results, our research showed that persister cells were present based on complete removal of isolates using amphotericin B 100 μg/ml, which was not observed in all *C. albicans* isolates (LaFleur et al., 2010). The reason for this difference in the presence and level of persister cells in some isolates and their absence in others is widely discussed and challenged among microbiological researchers (Bink et al., 2011; Stewart and Rozen, 2012). Some researchers have suggested that this difference may be due to the high prevalence of persister, its evolution, and resistance to adverse and stressful conditions (Balaban et al., 2004; Levin et al., 2014). According to a study on persister cells of microorganisms, this group of patients is prone to infections, immune system factors, and antibiotics due to low CD4 counts and receiving immunosuppressive drugs, and they are not able to completely eliminate the pathogen. As a result, these factors can cause relapse and recurrence of the disease (Cohen et al., 2013; Patra and Klumpp, 2013; Van den Bergh et al., 2017). It is argued that persister cells are a type of population-based defense tactic counteracting environmental change and stress (Kussell et al., 2005; Veening et al., 2008). Our findings showed that there is a high rate of persister in patients undergoing chemotherapy as well as HIV patients, which is consistent with the study by LaFleur et al. (2010). The results showed that the expression of BCR1 in the persister group in inducing conditions has an increase of about 10 times compared to the non-persister group. Promoting conditions fully confirmed the results of inducing conditions in that persister isolates have the potential to further increase the expression of *BCR1* and biofilm formation. These data suggested that higher biofilm formation is due to persister cells causing survival against drug treatment in *C. albicans* isolates. Our findings were consistent with the study by Li et al. (2018) on patients with persister and non-persister candidiasis, and *C. albicans* isolates with persister cells had a higher biofilm intensity. *BCR1* gene is essential for biofilm formation in the laboratory as well as in animal models with catheter-based candidiasis. BCR1 proteins express major surface adhesins such as HWP1 and ALS3,1, and *ALS3,1* genes are BCR1-dependent (Nobile and Mitchell, 2005; Dwivedi et al., 2011; Finkel et al., 2012). These reports and our results show that the surface adhesion step in the biofilm, which is controlled by BCR1 gene, plays a critical role in the production of persister and that the formation of the persister depends on the surface adhesion. The study by Sun et al. (2016) examined the production of biofilm in different phases and showed that persister are mainly produced in adhesion and biofilm formation phases. In a 2020 study, vaginal candidiasis was detected in a mouse model using *C. albicans* wild-type isolate and *bcr1*ΔΔ mutant, which reported the *BCR1* gene as a recurrence of the disease, and our data are consistent with this study (Wu et al., 2020).

In our research, the results of *EFG1* expression in the two groups of persister and non-persister showed that there was no significant difference between the two groups in inducing and promoting conditions. *EFG1* regulates morphology (yeast transformation to hyphae) and biofilm development in *C. albicans* (Lassak et al., 2011). Our results showed that biofilm expansion has no role in the production of persister cells and that persister cell production does not require the formation of a complex biofilm structure. LaFleur et al. (2006) reported that *efg*Δ*/cph1*Δ mutants were able to produce persister, which is consistent with our results. Therefore, according to the gene expression reports of *EFG1* and *BCR1* biofilm production pathways in this study, it can be concluded that the most important factor in the formation of persister is surface adhesion and biofilm formation, not biofilm development or mature biofilm production. This study suggests that the design of an anti-biofilm drug targeting the *BCR1* can eliminate biofilms and prevent the formation of persister cells in people with mucosal candidiasis or those with candidiasis from venous catheters, dentures, and implants.

The results of *CAT1* in inducing and promoting conditions in two groups of persister and non-persister cells showed that increasing expression of catalase enzyme has a significant role in the production of persister. Our results are consistent with other studies on oxidative stress response pathways on *SOD* and *AHP1*, both of which play a role in the production of persister in *C. albicans* isolates (Bink et al., 2011; Truong et al., 2016). The mRNA level of catalase strongly increases against the oxidative stress response in *C. albicans*. Catalase is among the limited antioxidants secreted outside and inside the cell, and in this respect, it acts differently from other antioxidants of oxidative stress response, except for the rapid response and immediate detoxification of H_2_O_2_, and over time, its amount in the cell regulates and reduces the ROS signal. Thus, catalase plays a major role in the rapid regulation of the response to oxidative stress after exposure to H_2_O_2_ (Nakagawa, 2008; Komalapriya et al., 2015; Pradhan et al., 2017). These results highlighted the key role of catalase in this pathway, and recent reports and studies highlight the importance of the oxidative stress response and the role of catalase in the production of persister cells. This relationship can be effective and practical for controlling persister formation in fungal biofilms.

Our findings regarding the phylogenetic tree in persister and non-persister cells showed that there is little difference between them and that the affinity between persister and non-persister cells is high. Consequently, it is suggested that the changes in nucleotides may be the root cause of persister cells. Therefore, according to the results of the evolutionary process in other microorganisms as well as our study, persister cells are phenotypic wild-type variants. Presumably, long-term and high-dose treatment with lethal antibiotics can determine the progression of persister cell formation (Mulcahy et al., 2010; Van den Bergh et al., 2017).

## Conclusion

Our findings show that there are small subsets of cells tolerating high doses of fungicidal compounds among *C. albicans* biofilm-forming isolates from chemotherapy and HIV patients, which are called persister cells. The high presence of persister cells in this group of patients may be due to prophylaxis, frequent exposure to antibiotics, and a defective immune system. Molecular analysis of two important genes in the biofilm production pathway showed that biofilm production and binding play an important role in the production of persister cells and that the catalase gene of the oxidative stress response pathway can be mentioned as a therapeutic target for the removal of persister cells. However, the conversion of yeast to hyphae and the maturation of biofilm do not affect the formation of persister.

## Data Availability Statement

The datasets presented in this study can be found in online repositories. The names of the repository/repositories and accession number(s) can be found below: DDBJ, access number: LC612887-612906.

## Ethics Statement

The studies involving human participants were reviewed and approved by the ethical code# IR.AJUMS.REC.1397.894. Written informed consent to participate in this study was provided by the participants’ legal guardian/next of kin.

## Author Contributions

MF was involved in the study design and interpretation of the data of the study, and the final editing of the manuscript. EA contributed to all the steps of experimental work, data analysis, and preparation of the manuscript draft. MT contributed to the interpretation and analysis of the data. MH contributed to the collection and preparation of clinical samples. All authors contributed to the article and approved the submitted version.

## Conflict of Interest

The authors declare that the research was conducted in the absence of any commercial or financial relationships that could be construed as a potential conflict of interest.

## Publisher’s Note

All claims expressed in this article are solely those of the authors and do not necessarily represent those of their affiliated organizations, or those of the publisher, the editors and the reviewers. Any product that may be evaluated in this article, or claim that may be made by its manufacturer, is not guaranteed or endorsed by the publisher.

## Footnotes

1
https://www.ebi.ac.uk/goldman-srv/webprank/

## Supplementary Material

Supplementary Figure 1 The Mean of 2^–Δct–^of *CAT1, BCR1*, and *EFG1* genes in persister and non-persister isolates in inducing and promoting conditions (*t*-test, **P*-value < 0.05, ***P*-value < 0.01).

Supplementary Table 1 Demographic information of HIV patients with and without persister cells. ^a^The test was performed after pooling the last two groups (Elementary education, Higher education) in order to eliminate small expected frequencies (Fisher’s exact test, *P*-value < 0.05).

Supplementary Table 2 Quantitative information about HIV patients in the two groups of persister and non-persister cell. Data are median (IQR) or Mean ± SD. ^∗^*P*-value < 0.05, (*t*-test and Mann–Whitney test).

Supplementary Table 3 Information about patients undergoing chemotherapy in the two groups of persister and non-persister cells. Data are median (IQR) or Mean ± SD (*t*-test and Mann–Whitney test), *P*-value < 0.05.
