# Supplementary figures and images for: Transcription Factors of CAT1, EFG1, and BCR1 Are Effective in Persister Cells of Candida albicans-Associated HIV-Positive and Chemotherapy Patients
Source: Front Microbiol. 2021 Aug 24;12:651221. doi: 10.3389/fmicb.2021.651221 (PMC8425484; doi:10.3389/fmicb.2021.651221)

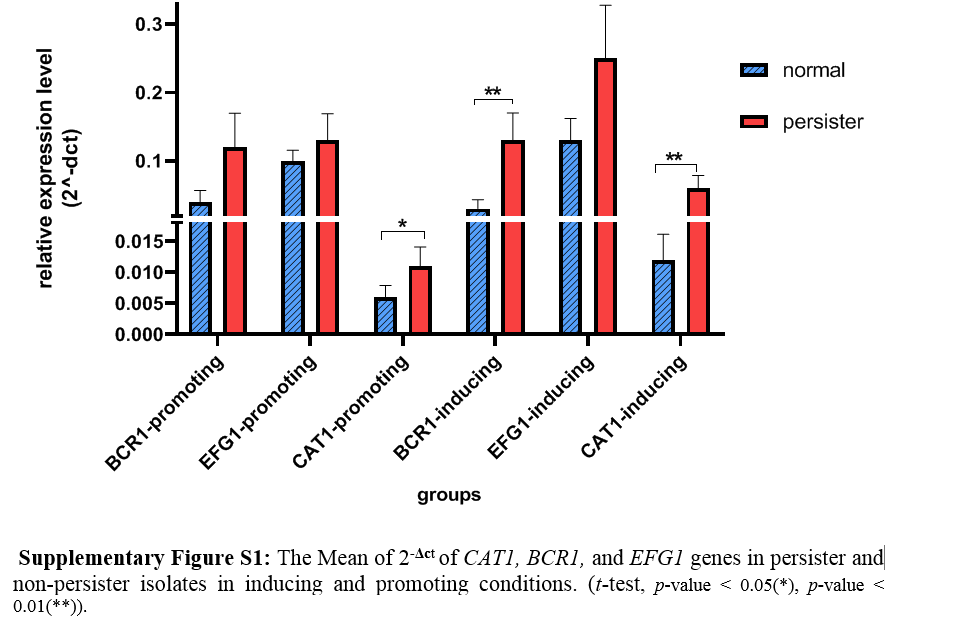

Supplement: Supplementary Figure 1 — The Mean of 2–Δct–of CAT1, BCR1, and EFG1 genes in persister and non-persister isolates in inducing and promoting conditions (t-test, *P-value < 0.05, **P-value < 0.01). [file Image_1.tiff]
