# Supplementary material for: Transcription Factors of CAT1, EFG1, and BCR1 Are Effective in Persister Cells of Candida albicans-Associated HIV-Positive and Chemotherapy Patients
Source: Front Microbiol. 2021 Aug 24;12:651221. doi: 10.3389/fmicb.2021.651221 (PMC8425484; doi:10.3389/fmicb.2021.651221)
Supplement: Supplementary Table 1 — Demographic information of HIV patients with and without persister cells. aThe test was performed after pooling the last two groups (Elementary education, Higher education) in order to eliminate small expected frequencies (Fisher’s exact test, P-value < 0.05). [file Table_1.docx]

Supplementary Table S1: Demographic information of HIV patients with and without persister cells

| P Value^*^ | Variable none N (%) low N (%) | | |
| --- | --- | --- | --- |
| Gender | | | |
| .708 | 36(72.0) | 6(66.7) | Male |
|  | 14(28.0) | 3(33.3) | Female |
| Drug injection | | | |
| .277 | 29(58.0) | 3(33.3) | True |
|  | 21(42.0) | 6(66.7) | False |
|  | TB | | |
| .641 | 8(16.0) | 2(22.2) | Yes |
|  | 42(84.0) | 7(77.8) | No |
| TB_Prophylaxis | | | |
| 1.000 | 5(10.0) | 0(0.0) | Yes |
|  | 45(90.0) | 9(100.0) | No |
| PCP | | | |
| 1.000 | 7(14.0) | 1(11.1) | Positive |
|  | 43(86.0) | 8(88.9) | Negative |
| ARV | | | |
| 1.000 | 48(96.0) | 9(100.0) | Yes |
|  | 2(4.0) | 0(0.0) | No |
| Receiving_Blood | | | |
| 0.153 | 0(0.0) | 1(11.1) | True |
|  | 50(100.0) | 8(88.9) | False |
| Mother_to_Child | | | |
| 1.000 | 2(4.0) | 0(0.0) | True |
|  | 48 (96.0) | 9(100.0) | False |
| Addiction_History | | | |
| 0.277 | 29(58.0) | 3(33.3) | Yes |
|  | 21(42.0) | 6(66.7) | No |
| Education | | | |
| 0.288^a^ | 5(10.0) | 2(22.2) | No education |
|  | 45 (90.0) | 5(55.6) | Elementary education |
|  | 0(0.0) | 2(22.2) | Higher education |
| Homosexuality_ | | | |
| 0.494 | 3(6.0) | 1(11.1) | True |
|  | 47 (94.0) | 8(88.9) | False |
| HCV_Ag | | | |
| 1.000 | 16(32.0) | 3(33.3) | Positive |
|  | 34 (68.0) | 6(66.7) | Negative |
| HBS_Ag | | | |
| 0.397 | 2(4.0) | 1(11.1) | Positive |
|  | 48 (96.0) | 8(88.9) | Negative |
| Occupational_Exposure | | | |
| - | 0(0.0) | 0(0.0) | True |
|  | 50 (100.0) | 9(100.0) | False |
| Common_Injection_History | | | |
| 0.725 | 21(42.0) | 3(33.3) | Yes |
|  | 29(58.0) | 6(66.7) | No |
| CD4 | | | |
| 1.000 | 10(20.0) | 2(22.2) | <200 |
|  | 40(80.0) | 7(77.8) | >200 |

^a^ :The test was performed after pooling the last two groups (Elementary education, Higher education) in

order to eliminate small expected frequencies (Fisher's exact test, P-value <0.05)
