# Supplementary material for: Transcription Factors of CAT1, EFG1, and BCR1 Are Effective in Persister Cells of Candida albicans-Associated HIV-Positive and Chemotherapy Patients
Source: Front Microbiol. 2021 Aug 24;12:651221. doi: 10.3389/fmicb.2021.651221 (PMC8425484; doi:10.3389/fmicb.2021.651221)
Supplement: Supplementary Table 2 — Quantitative information about HIV patients in the two groups of persister and non-persister cell. Data are median (IQR) or Mean ± SD. ∗P-value < 0.05, (t-test and Mann–Whitney test). [file Table_2.docx]

Supplementary Table S2: Quantitative information about HIV patients in the two groups of persister and non persister cell

| **Variable** | **None** | | **Low** | | **P Value** |
| --- | --- | --- | --- | --- | --- |
|  | N (19%) | Mean±SD | N (81%) | Mean±SD |  |
| **Age** | 9 | **40.33±5.40** | 50 | **40.36±14.47** | 0.996 |
| **Viral load** | 9 | **79.00(.00-455.10)** | 50 | **.00(.00-2195.67)** | **0.60** |
| **Candida-load** | 9 | **80.00(30.00-476.00)** | 50 | **68.50(19.50-120.00)** | 0.506 |
| **Biofilm** | 9 | **1.00(.77-2.80)** | 50 | **2.50(2.01-3.77)** | .021^*^ |

Data are median (IQR) or Mean±SD.* : P-value <0.05, ( t-test and Mann–Whitney test)
