# Supplementary material for: Transcription Factors of CAT1, EFG1, and BCR1 Are Effective in Persister Cells of Candida albicans-Associated HIV-Positive and Chemotherapy Patients
Source: Front Microbiol. 2021 Aug 24;12:651221. doi: 10.3389/fmicb.2021.651221 (PMC8425484; doi:10.3389/fmicb.2021.651221)
Supplement: Supplementary Table 3 — Information about patients undergoing chemotherapy in the two groups of persister and non-persister cells. Data are median (IQR) or Mean ± SD (t-test and Mann–Whitney test), P-value < 0.05. [file Table_3.docx]

Supplementary Table S3: Information about patients undergoing chemotherapy in the two groups of persister and non-persister cells

| **Variable** | **None** | | **L0W** | | **P Value** |
| --- | --- | --- | --- | --- | --- |
|  | N (38%) | Mean±SD | N (62%) | Mean±SD |  |
| **Age** | 12 | 56.25±12.01 | 27 | 56.62±15.21 | .940 |
| **Candida-load** | 12 | 245.00(46.25-11750.00) | 27 | 140.00(40.00-280.00) | .229 |
| **Biofilm** | 12 | 3.80(2.05-4.80) | 27 | 2.40(1.40-4.40) | .185 |

Data are median (IQR) or Mean±SD.( t-test and Mann–Whitney test), P-value <0.05
